# Supplementary material for: Mediating-Moderating Effect of Allostatic Load on the Association between Dietary Approaches to Stop Hypertension Diet and All-Cause and Cause-Specific Mortality: 2001–2010 National Health and Nutrition Examination Surveys
Source: Nutrients. 2019 Sep 29;11(10):2311. doi: 10.3390/nu11102311 (PMC6836046; doi:10.3390/nu11102311)
Supplement: Supplementary file 1 [file nutrients-11-02311-s001.pdf]

Table 1. DASH diet scoring system.

Nutrient	DASH diet nutrient composition [a]	DASH score target (1)	Intermediate target (0.5)
Saturated fat	6% of energy	6% of energy	11% of energy
Total fat	27% of energy	27% of energy	32% of energy
Protein	18% of energy	18% of energy	16.5% of energy
Cholesterol	150 mg	71.4 mg/1000 kcal	107.1 mg/1000 kcal
Fiber	31 g	14.8 g/1000 kcal	9.5 g/1000 kcal
Magnesium	500 mg	238 mg/1000 kcal	158 mg/1000 kcal
Calcium	1240 mg	590 mg/1000 kcal	402 mg/1000 kcal
Potassium	4700 mg	2238 mg/1000 kcal	1534 mg/1000 kcal
Sodium [b]	2400 mg	1143 mg/1000 kcal	1286 mg/1000 kcal

Abbreviation: DASH, Dietary Approaches to Stop Hypertension trial. [a] Based on a 2100-kcal diet. [b] Sodium target based on the Sixth Report of the Joint National Committee on Prevention, Detection, Evaluation, and Treatment of High Blood Pressure recommendations.

Source:[1]

1. Mellen PB, Gao SK, Vitolins MZ, Goff DC, Jr. Deteriorating dietary habits among adults with hypertension: DASH dietary accordance, NHANES 1988-1994 and 1999-2004. Archives of internal medicine 2008;168:308-14.

Table 2. Allostatic Load Score.

	1	0.5	0
**SBP**	≥150 mmHg	120 to <150 mmHg	<120 mmHg
**DBP**	≥90 mmHg	80 to <90 mmHg	<80 mmHg
**BMI**	≥30 kg/m^2	25 to <30 kg/m^2	<25 kg/m^2
**HbA1c**	≥6.5%	5.7% to <6.5%	<5.7%
**Total cholesterol**	≥240 mg/dL	200 to <240 mg/dL	<200 mg/dL
**HDL cholesterol**	<40 mg/dL	40 to <60 mg/dL	≥60 mg/dL
**Total/HDL cholesterol ratio**	≥6	5 to <6	<5
**CRP**	≥3 mg/L	1 to <3 mg/L	<1 mg/L
**Albumin**	<3.0 μg/mL	3.0 to <3.8 μg/mL	≥3.8 μg/mL
**Creatinine clearance**	<30 mL/min/1.73 m^2	30 to <60 mL/min/1.73 m^2	≥60 mL/min/1.73 m^2

Source:[2]

2. Rodriquez EJ, Livaudais-Toman J, Gregorich SE, Jackson JS, Napoles AM, Perez-Stable EJ. Relationships between allostatic load, unhealthy behaviors, and depressive disorder in U.S. adults, 2005-2012 NHANES. Preventive medicine 2018;110:9-15.
